# Single-Cell Transcriptomics Unveils the Dedifferentiation Mechanism of Lung Adenocarcinoma Stem Cells

**DOI:** 10.3390/ijms24010482

**Published:** 2022-12-28

**Authors:** Zhenhua Pan, Meidi Zhang, Fengyu Zhang, Hongli Pan, Yongwen Li, Yi Shao, Xin Yuan, Ju Wang, Jun Chen

**Affiliations:** 1 Tianjin Key Laboratory of Lung Cancer Metastasis and Tumor Microenvironment, Tianjin Lung Cancer Institute, Tianjin Medical University General Hospital, Tianjin 300052, China; 2 School of Biomedical Engineering, Tianjin Medical University, Tianjin 300070, China; 3 Department of Lung Cancer Surgery, Tianjin Lung Cancer Institute, Tianjin Medical University General Hospital, Tianjin 300052, China

**Keywords:** lung adenocarcinoma, serum-free cell culture, scRNA-seq, cancer stem cells, dedifferentiation

## Abstract

Lung adenocarcinoma (LUAD) is a major subtype of lung cancer, and its prognosis is still poor due to therapy resistance, metastasis, and recurrence. In recent years, increasing evidence has shown that the existence of lung cancer stem cells is responsible for the propagation, metastasis, therapy resistance, and recurrence of the tumor. During their transition to cancer stem cells, tumor cells need to inhibit cell differentiation and acquire invasive characteristics. However, our understanding of the property and role of such lung cancer stem cells is still limited. In this study, lung adenocarcinoma cancer stem cells (LCSCs) were enriched from the PC-9 cell line in a serum-free condition. PC-9 cells grew into spheres and showed higher survival rates when exposed to gefitinib: the drug used for the treatment of LUAD. Additionally, we found that the canonical stemness marker protein *CD44* was significantly increased in the enriched LCSCs. Then, LCSCs were inoculated into the groin of nude mice for 1.5 months, and tumors were detected in the animals, indicating the strong stemness of the cells. After that, we performed single-cell RNA sequencing (scRNA-seq) on 7320 LCSCs and explored the changes in their transcriptomic signatures. We identified cell populations with a heterogeneous expression of cancer stem marker genes in LCSCs and subsets with different degrees of differentiation. Further analyses revealed that the activation of the *FOXM1* (oncoprotein) transcription factor is a key factor in cell dedifferentiation, which enables tumor cells to acquire an epithelial-mesenchymal transition phenotype and increases the LCSC surface marker *CD44*. Moreover, we found that the combination of *CD44*, *ABCG2*, and *ALCAM* was a specific marker for LCSCs. In summary, this study identified the potential factors and molecular mechanisms underlying the stemness properties of LUAD cancer cells; it could also provide insight into developing novel and effective therapeutic approaches.

## 1. Introduction

Lung cancer is the second most common cancer worldwide, with 2.2 million new cases diagnosed each year. It remains the leading cause of cancer-related death and is responsible for about 1.8 million deaths annually [1]. Among the lung cancer cases, non-small cell lung cancer (NSCLC) and small cell lung cancer (SCLC) account for 85% and 15%, respectively [2]. Lung adenocarcinoma (LUAD) is the most common subtype of NSCLC, accounting for about 40% of all lung malignancies [3]. Despite the rapid development of new therapies, such as targeted therapy and immunotherapy, the prognosis of the disease is poor, with 5-year survival less than 15% [4,5,6,7,8]. The low survival rate is largely caused by the emergence of resistance prior to and during treatment, which represents one of the major challenges in lung cancer treatment and is the main factor responsible for the progression, metastasis, and recurrence of the disease [9,10,11].

In recent years, studies have demonstrated that intratumor heterogeneity may be the main reason for the difference in therapeutic sensitivity and low survival rate in cancer treatment. Intratumor heterogeneity refers to the coexistence of cells of distinct phenotypic and molecular features within a tumor and is a sustained obstacle to cancer therapy [12]. Studies have shown that lung cancer cells can obtain stem characteristics through dedifferentiation [13]. It is suggested that identifying lung cancer stem cells and their roles in tumor biology and treatment resistance may lead to better-targeted therapies for lung cancer [11]. However, the molecular features and mechanisms related to the dedifferentiation of lung adenocarcinoma cancer stem cells (LCSCs) still need to be well studied. 

The detection of cancer stem cell markers and the molecular mechanism they are involved in can guide the subsequent treatment of tumors and the evaluation of prognosis. However, because ordinary tumor cells can be dedifferentiated into cancer stem cells through genome changes, it is extremely difficult to identify cancer stem cells accurately. So far, a number of markers have been identified for LCSCs, such as *CD44*, *ABCG2*, *CD133*, *EpCAM*, and *ALCAM* [14,15,16]. It was also found that the abnormal activation of wnt/β-catenin, notch, and hedgehog signaling pathways is involved in the differentiation and dedifferentiation of cancer stem cells [17,18,19,20,21]. However, our understanding of the details of LCSCs, such as their origin, molecular characterization, and roles, is still incomplete [22]. 

In this study, we explored the features of LCSCs and the potential mechanisms underlying their dedifferentiation based on PC-9 cells, a LUAD cell line with *EGFR* mutation, and identified the prognostic biomarkers in the cell line. Briefly, earlier studies have detected a small group of cells with stem cell characteristics in the PC-9 cell line [23]; we obtained enriched LCSCs from PC-9 cells by the classic serum-free (SF) ultra-low adsorption spheroidization enrichment method. Subpopulations with a heterogeneous expression of LCSC marker genes and different degrees of differentiation in enriched cells were identified through scRNA-seq analyses. Then, we found that the combined expression of classical cancer stem markers (*CD44*, *ABCG2*, and *ALCAM*) can be used to predict the prognosis of LUAD. Further analyses revealed that the activation of *FOXM1* is a key factor in cell dedifferentiation and anti-apoptotic. Altogether, our study revealed a mechanism that involved a balance between LCSC renewal and differentiation, which may be important in promoting the malignant tumor state and tumor stemness of LCSCs.

## 2. Results

### 2.1. Sphere-Forming Culture Enriching Stem Cells of PC-9 Cell Line

**Figure 1:**
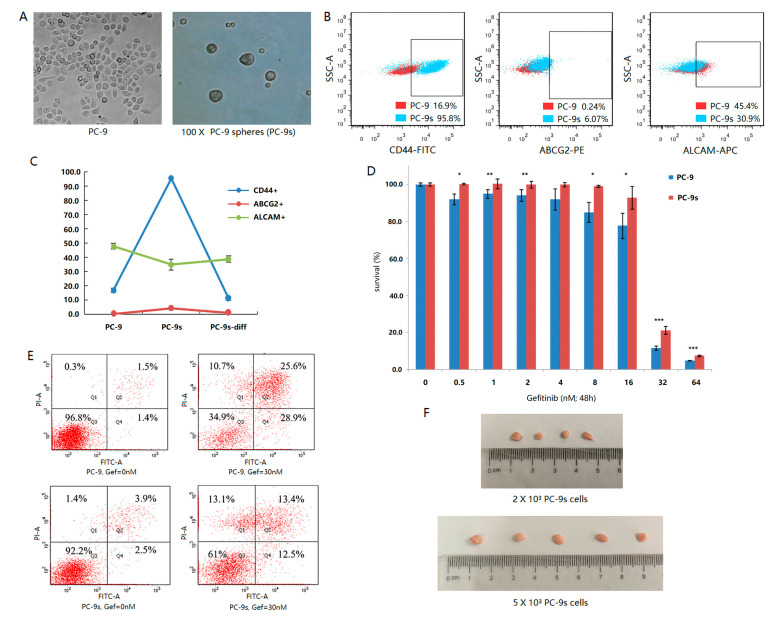
Under the microscope, PC-9 adherent cells and PC-9s cell spheres are enriched by 100 times magnification (**A**). The surface antigen fluorescence intensity of *CD44*, *ABCG2*, and *ALCAM* of PC-9 cells and PC-9s cells were compared by flow cytometry. Red points represent PC-9 cells and blue points represent PC-9s cells. The horizontal axis is the intensity of antigen fluorescence, and the vertical axis is the intensity of lateral light (**B**). The fluorescence intensity of *CD44* (blue), *ABCG2* (red), and *ALCAM* (green) surface antigen was detected by flow cytometry. PC-9s-diff are PC-9s cells after one week of re-adherent. The vertical axis represents the percentage of positive cells (**C**). The relative survival rate of cells treated with different doses of gefitinib (nM) for 48 h (0 dose group as control). The differences of cell survival rates were calculated by Wilcox test (*p*-value < 0.05: *, *p*-value < 0.01: **, *p*-value < 0.001: ***) (**D**). Apoptotic level of 30 nM gefitinib after 48 h. The horizontal axis is the intensity of Annexin-FITC fluorescence, and the vertical axis is the intensity of PI fluorescence (**E**). PC-9s cells of different doses (up: 2000 cells/animal, down: 5000 cells/animal) were inoculated subcutaneously into the groin of nude mice and dissected after 1.5 months (**F**).

In this study, we used the human lung adenocarcinoma PC-9 cell line as the model of LCSCs. PC-9 cells were cultured by the serum-free low adsorption globular enrichment method, and stable tumor spheres were obtained in 4 weeks, which are referred to as PC-9s below (Figure 1A). Then, the degree of stemness of the PC-9 and PC-9s cells was compared based on their mRNAsi scores, which were calculated from the bulk RNA-seq data corresponding to PC-9 and PC-9s cells. The mRNAsi score was significantly higher in PC-9s than in PC-9 (*p*-value = 0.038), indicating that PC-9s had stronger stemness than PC-9 (Table S1). The stemness of PC-9 and PC-9s cells were further analyzed via flow cytometry by checking the stemness marker proteins in PC-9 and PC-9s, including *CD44*, *CD133*, *ABCG2*, *EpCAM*, and *ALCAM*. The results showed that the positive expression of *CD44* and *ABCG2* increased with the increase in enrichment time, especially for *CD44*. However, the positive expression of *ALCAM* decreased with the increase in enrichment time (Figure 1B). We further compared the sensitivity of PC-9s cells and PC-9 cells to gefitinib. The results showed that the survival rate of PC-9s was significantly higher than that of PC-9 cells, and apoptosis was significantly reduced under the same doses of gefitinib (Figure 1D,E). After the PC-9s and PC-9 cells were inoculated into the groin of nude mice for 1.5 months, the tumors were dissected from mice inoculated with PC-9s cells, while no tumor was found in those inoculated with PC-9 cells (Figure 1F). To further test the differentiation ability of PC-9s cells, we reattached the enriched cells and routinely cultured them. After one week of adherence, the stemness markers on the cell surface returned to a level similar to that of parental PC-9 cells (Figure 1C).

### 2.2. Single-Cell Transcriptomics Reveals Heterogeneity of Stem Subclones within PC-9s

**Figure 2:**
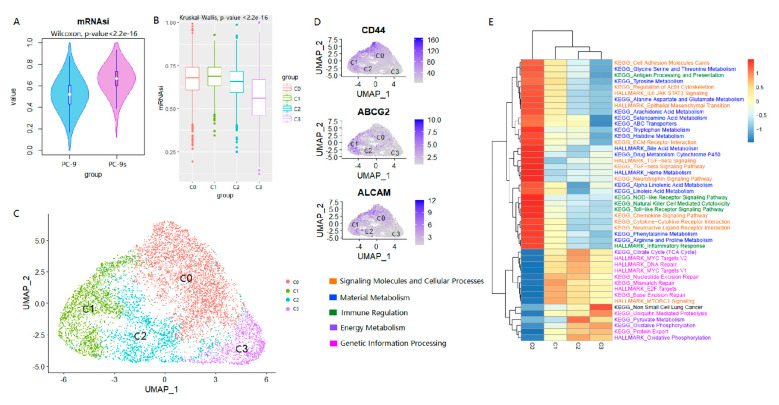
The heterogeneity of stem cells in PC-9s. The mRNAsi values of cells of cell line PC-9 were lower than cell line PC-9s significantly (Wilcox test, *p*-value < 2.2 × 10^−16^) (**A**). The mRNAsi scores of the four cell clusters were decreased significantly and gradually, although there was a small increase in C1 (Kruskal–Wallis, *p*-value < 2.23 × 10^−16^) (**B**). UMAP projection of 7320 cells from cell line PC-9s. Different cell clusters are colored with unique colors: C0 (45.5%, 3327 cells), C1 (24.6%, 1801 cells), C2 (17.1%, 1250 cells), and C3 (12.8%, 942 cells) (**C**). UMAP projections of cell line PC-9s show the gene expression of three common LCSC markers (*CD44*, *ABCG2*, *ALCAM*) in different cell clusters (C0, C1, C2, and C3). The darker the color of the dot, the higher the gene expression (**D**). GSVA analysis of KEGG and HALLMARK for four cell subpopulations. Different colors indicate different kinds of entries, including signaling molecules and cellular processes, material metabolism, immune regulation, energy metabolism, and genetic information processing (**E**).

The results of the flow cytometry above show that the enriched PC-9s cells had heterogeneous characteristics, such as the variation in the number of cells within and between different stem marker protein-positive groups. To further explore whether the coexistence of LCSCs and primary LUAD cells, as well as their mutual transformation, was related to the intratumor heterogeneity, we analyzed the scRNA-seq data of PC-9s cells. As demonstrated above, PC-9s had stronger stemness than PC-9 cells in our cell cultures, which was further confirmed via the scRNA-seq dataset GSE149383 measured for PC-9 and PC-9s cells. After quality control and filtration, 10,716 PC-9 cells and 7320 PC-9s cells were retained (Materials and Methods). In line with the results shown above, we found that the mRNAsi scores of PC-9s cells were also significantly higher than PC-9 cells at the single-cell level (Wilcox test, *p*-value < 2.2 × 10^−16^; Figure 2A).

To explore whether the LCSCs were heterogeneous, the stemness index difference among PC-9s cells was examined. The 7320 PC-9s cells were clustered into four major clusters, i.e., C0 (3327 cells, 45.5%), C1 (1801 cells, 24.6%), C2 (1250 cells, 17.1%), and C3 (942 cells, 12.8%) (Figure 2C). To determine the differences at the gene expression level among these four cell clusters, we obtained the DEGs for each cluster (Table S2). We found that each of the three common LCSC markers (i.e., *CD44*, *ABCG2*, *ALCAM*) was significantly up-regulated in one of the cell clusters and significantly downregulated in other clusters, i.e., *CD44*+*ABCG2*-*ALCAM*- in C0, *CD44*-*ABCG2*+*ALCAM*- in C1, and *CD44*-*ABCG2*-*ALCAM*+ in C2 and no common LCSC markers were detected in C3 (Figure 2D, Table S2). Thus, the combinations of these LCSC markers were specific in each PC-9s cell cluster. At the same time, we found that the mRNAsi scores decreased gradually from C0 to C3 (Kruskal–Wallis test, *p*-value < 2.2 × 10^−16^), with C3 having the lowest score (Figure 2B), indicating that the cells in these clusters were from different populations. Additionally, such results indicated that compared with other clusters, the cells in C3 might be relatively more differentiated.

We further analyzed the functional features of the differentially expressed genes in these four cell subpopulations (Figure 2E). First, we noticed that the characteristics of epithelial-mesenchymal transition (EMT) in the four groups gradually decreased from C0 to C3, while the characteristics of non-small cell lung cancer gradually increased. In addition, the genes with gradually decreased GSVA scores were mainly involved in metabolisms, such as amino acids, signaling molecules, cellular processes (cell adhesion, cytokine-cytokine receptor interaction, etc.), and immune regulation such as antigen processing and presentation. Compared with the other three cell subpopulations, the activity of cellular signal pathways in the C0 subpopulation was significantly increased, especially the pathways related to inflammatory response and growth factor, which were higher than that of other subpopulations. It should be noted that the metabolic pathways in C0 cells were activated, including ABC transporters and drug metabolism cytochrome P450.

For the C1 cluster, the mRNAsi scores were similar to that of C0 and were significantly higher than the other two cell subgroups. Their stemness was characterized by the upregulation of the *ABCG2* transcription level. From the perspective of transcriptome characteristics, the DNA repair of C1 cells was significantly higher than those of the other three cell subgroups, including mismatch repair, base excision repair, E2F targets, mTOR downstream pathway, etc. C2 also showed strong DNA repair and proliferation ability, including DNA repair and MYC target gene activation. At the same time, the C2 cell subpopulation showed the activation of oxidative phosphorylation, citrate cycle, pyruvate metabolism, etc., reflecting the characteristics of vigorous energy metabolism during rapid cell proliferation.

### 2.3. The Combination of CD44, ABCG2 and ALCAM Is Confirmed in the Clinical Prognosis of LUAD Patients

**Figure 3:**
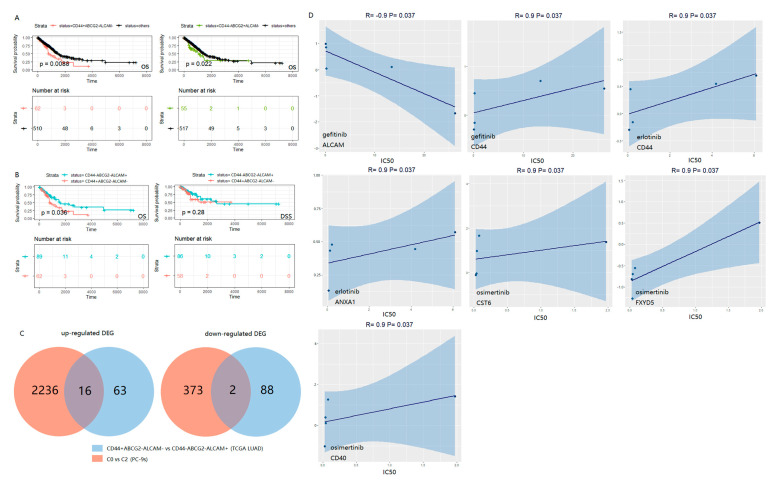
Survival curves (OS and DSS) of TCGA LUAD patients. High level (+) and low level (−) with the median of each marker gene expression across all patients as the threshold. The number corresponding to “time = 0” below the curves represent the number of patients with corresponding survival information (**A**,**B**). Venn plots show the number of intersected up-regulated DEGs and down-regulated DEGs of TCGA LUAD (*CD44*+*ABCG2*−*ALCAM*− vs. *CD44*−*ABCG2*−*ALCAM*+) and PC-9s (C0 vs. C2), respectively (**C**). Regression graphs display the Spearman correlation of EGFR-TKIs drug sensitivity and gene expression in all five EGFR mutant cell lines (NCI-H1650, NCI-H1975, NCI-H1975, PC-14, PC-3, and HCC-827) in GDSC2 (https://www.cancerrxgene.org/; accessed on 24 June 2022). Positive correlation indicates drug resistance, and negative correlation indicates drug sensitivity (**D**).

To validate the prognostic value of the three LCSC markers which we identified, the gene expression and clinical data of 585 LUAD patients retrieved from TCGA were used as a test set. The patients were divided into a high-expression group and a low-expression group by using the median of the expression of each marker gene across all patients as the threshold. Then, the prognosis significance of these markers was compared in the patient groups (i.e., *CD44*+/−, *ABCG2*+/−, and *ALCAM*+/−). Patients with marker *CD44*+*ABCG2*-*ALCAM*- were associated with a poor prognosis (*p*-value = 0.0088), and a similar correlation was detected in patients with *CD44*-*ABCG2*+*ALCAM*- (*p*-value = 0.022) (Figure 3A). As shown earlier, at the single-cell level, the C0 cluster (*CD44*+*ABCG2*-*ALCAM*-) and C1 cluster (*CD44*-*ABCG2*+*ALCAM*-) in Figure 2 tended to have higher mRNAsi scores. Thus, the poor prognosis in patients with marker *CD44*+*ABCG2*-*ALCAM*- or *CD44*-*ABCG2*+*ALCAM*- might be related to the more active biological processes in LCSCs and greater tumor dedifferentiation. At the same time, we analyzed the survival patterns of other marker combinations (e.g., *CD44*+*ABCG2*+*ALCAM*+) and patient groups; none of them was associated with a significant prognostic effect (Figure S2). So, the dimensionality reduction and clustering of our single-cell data were meaningful and traceable to separate the three common LCSC markers.

Moreover, compared to the combined marker *CD44*−*ABCG2*−*ALCAM*+, marker *CD44*+*ABCG2*−*ALCAM*− was associated with decreased overall survival (OS) (*p*-value < 0.05) (Figure 3B). In addition, *CD44*+*ABCG2*−*ALCAM*− was related to decreased disease-specific survival (DSS) compared with *CD44*−*ABCG2*−*ALCAM*+ in the first five years. These observations were consistent with the results of the stemness analysis based on the mRNAsi scores of the C0 and C2 clusters (Figure 2C). We further investigated the role of ABCG2 by comparing different patients (e.g., *CD44*+*ABCG2*+*ALCAM*− and *CD44*−*ABCG2*+*ALCAM*+) (Figure S3A) and did not find that it was correlated with OS or DSS, indicating that ABCG2- might have a special prognostic value of the combination mode (Figure 3A).

These markers had similar properties in patients and cells. In the first place, the batch-effect correction of PC-9 and PC-9s was performed by the Seurat MultiCCA method (Methods, Figure S1A). Next, according to the expression of LCSC markers, we divided the cell population into two clusters after batch effect treatment (i.e., *CD44*+*ABCG2*−*ALCAM*− and *CD44*−*ABCG2*−*ALCAM*+) (Figure S1B,D). The mRNAsi scores of the cluster with CD44+ABCG2−ALCAM− were significantly higher than *CD44*−*ABCG2*−*ALCAM*+ (Wilcox test, *p*-value < 2.2 × 10^−16^; Figure S1C). In addition, the number of PC-9 cells was much larger than the PC-9s cells in the *CD44*−*ABCG2*−*ALCAM*+ cell cluster (PC-9: 99.9%, 10,710 cells; PC-9s: 67%, 4902 cells). In *CD44*+*ABCG2*−*ALCAM*−, the opposite was true (PC-9: 0.1%, 6 cells; PC-9s: 33%, 2418 cells). 

Then, we identified the differentially expressed genes between the TCGA LUAD groups (i.e., *CD44*+*ABCG2*−*ALCAM*− vs. *CD44*−*ABCG2*−*ALCAM*+) and the PC-9s clusters C0 vs. C2. In the two lists of genes, there were 16 up-regulated genes and two down-regulated genes that were commonly expressed in the patients and PC-9 cells (Figure 3C, Table S3). Among the up-regulated genes, seven genes (i.e., *AREG*, *CDA*, *CST6*, *FGFBP1*, *FXYD5*, *KRT6A*, and *KRT16*) were negatively correlated with the OS of all the TCGA LUAD patients analyzed (*p*-value < 0.05; Figure S3B). Since our PC-9s cell model was EGFR mutant, we further explored the performance of the screened genes in the sensitivity of EGFR-TKIs. Based on the correlation between the expression data of the screened DEGs in EGFR mutant LUAD cell lines and the drug sensitivity correlation of EGFR-TKIs in the GDSC2 database (https://www.cancerrxgene.org/; accessed on 24 June 2022) [24] (Methods), we found that CD44 was positively correlated with the IC_50_ of gefitinib and erlotinib. However, *ALCAM* was negatively correlated with the IC_50_ of gefitinib. This may explain why PC-9s show more survival rate in a gefitinib condition (Figure 3D).

### 2.4. Key Switches Affecting the Differentiation and Dedifferentiation of Cancer Stem Cells

**Figure 4:**
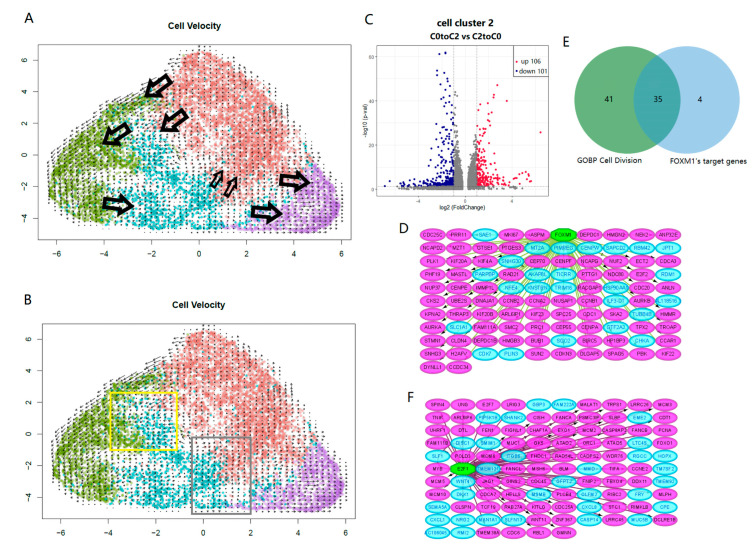
UMAP projection shows the RNA velocity of four clusters in PC-9s, with C0, C1, C2 and C3 shown in brown, green, cyan and purple, respectively; and the arrows indicate the transformation between cell clusters (**A**). In the C2 group, “C0 to C2” transformed cell subsets and “C2 to C0” transformed cell subsets were extracted. Cells in yellow frame were “C0 to C2” and cells in grey frame were “C2 to C0” (**B**). Volcano plot displays DEGs between “C0 to C2” and “C2 to C0”; there are 106 up-regulated DEGs in red points and 101 down-regulated DEGs in blue points (|log2FC| > 1 and *p*-value < 0.05) (**C**). Directed graphs show two main TFs in “C2 to C0” and “C0 to C2”, respectively (**D**,**F**). Green hexagons represent TFs, pink ellipses represent targeted genes, and blue ellipses represent other down-regulated DEGs (**D**) or up-regulated DEGs (**F**) that (**C**) mentioned. Venn diagram displays the intersection between core genes in GO BP cell division and FOXM1’s targeted genes (**E**).

To further explore the relationship between LCSC subpopulations, we performed RNA velocity analysis for the four clusters (i.e., C0-C3) in PC-9s. We observed that the developmental trajectory of C1 and C3 was relatively simple. For C0 and C2, there were not only the tracks of development from C0 to C2 but also the signs of development from C2 to C0 (Figure 4A). We extracted “C0 to C2” transformed cell subsets and “C2 to C0” transformed cell subsets from the C2 cluster. By comparing their transcriptome data, we identified 106 upregulated and 101 down-regulated DEGs (Figure 4B,C, Table S4). These genes were further analyzed by GSEA, and we found that the genes upregulated in “C0 to C2” were mainly enriched in pathways involved in the positive regulation of cell differentiation, DNA repair, and the regulation of immune response. However, genes upregulated in “C2 to C0” were primarily enriched in cell division and intracellular transport, etc. (Table S5).

To explore the direct key switches affecting the transformation relationship between C0 and C2, we enriched the TFs for DEGs between “C0 to C2” and “C2 to C0” and predicted the TFs affecting cell differentiation and dedifferentiation, respectively. In the genes upregulated in “C2 to C0”, FOXM1 TF was highly enriched (NES = 11.71). Combined with the result of GSEA, we found that 35 of the 39 targeted genes of *FOXM1* were core genes in cell divisions (Figure 4D,E). On the other hand, in the genes upregulated with “C0 to C2”, *E2F1* TF was highly enriched (NES = 8.16) and targeted 72 up-regulated DEGs in “C0 to C2” vs. “C2 to C0”. *E2F1* was also a core gene related to cell differentiation (Figure 4F, Table S5). 

### 2.5. Intercellular Communication Analysis Reveals the Sociality within PC-9s Cells

**Figure 5:**
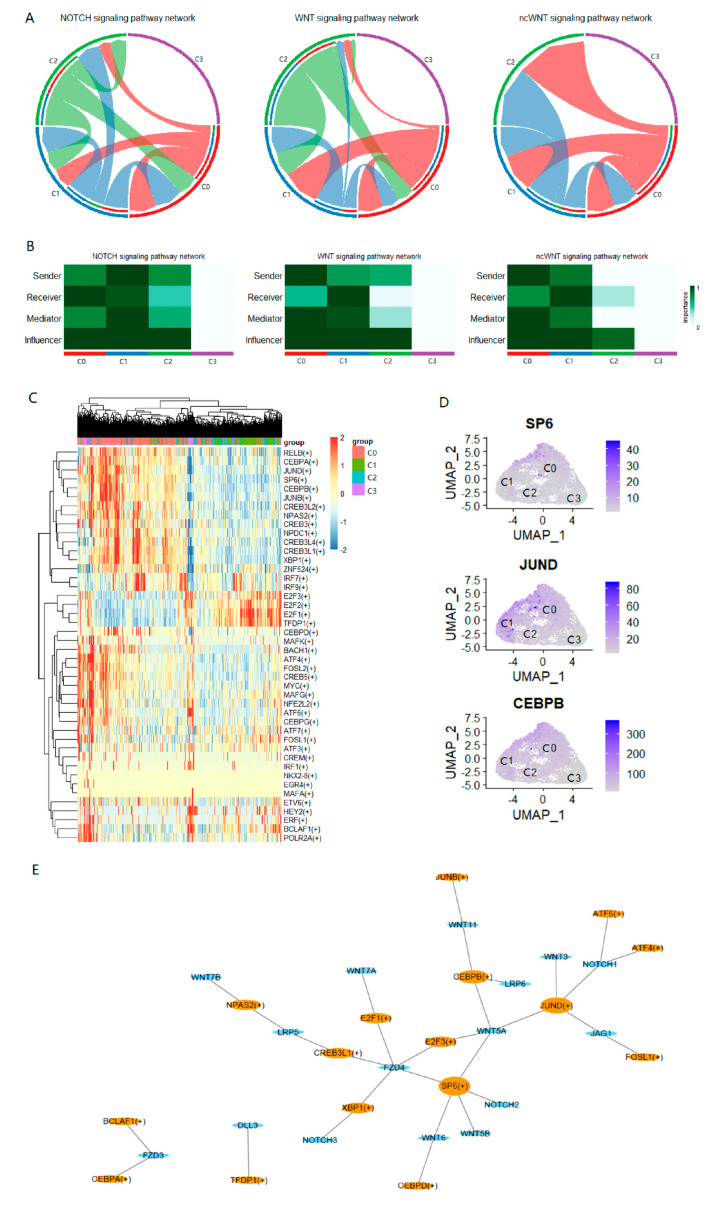
Ring diagram display pathway level interaction networks of NOTCH, WNT, and ncWNT. The arrow points to the signaling receiver and the other side is the signaling sender (**A**). Heat maps show the importance of roles including sender, receiver, mediator, and influencer. The darker the color, the more importanT it is (**B**). Heat map reveals the activation of predicted TFs (e.g., *RELB*(+)) in different cell clusters through SCENIC. Greater than 0 indicates activation and less than 0 indicates inhibition (**C**). UMAP projection of gene expression of three TFs in PC-9s: *SP6*, *JUND*, and *CEBPB* (**D**). The regulons, TFs (e.g., *SP6*(+)), and target genes about three signaling pathways. Orange ellipses represent TFs and blue ellipses represent target genes. The greater the degree, the larger the node (**E**).

While there was a clear dedifferentiation pattern of LCSCs at the cellular level, we further explored the molecular characteristics underlying the transformation of LCSCs in different differentiation states. Three common LCSC-related pathways were identified, i.e., NOTCH signaling, WNT signaling, and non-canonical WNT (ncWNT) signaling (Figure 5A). Among the four clusters of PC-9s, these signaling pathways were not enriched in cells in C3, and the expression of the genes in these pathways was lower (Figure S4B). Furthermore, we identified the roles of each cell cluster and found that C0 was the main receiver of the NOTCH signaling pathway and the main sender of WNT and ncWNT signaling pathways; C1 was the main sender of the NOTCH signaling pathway and the main receiver of WNT and ncWNT signaling pathways (Figure 5B). Comparing the three signaling pathways, we found that the ligand-receptor signal of WNT was stronger, and the ligand *WNT7B* and receptor *FZD4* + *LRP6* contributed more to the overall signaling pathway (Figure S4A). Finally, to identify the key factors influencing ligand-receptor signals, the predicted TFs related to the three LCSC pathways were obtained by matching the signaling pathways related to the genes obtained by cell–cell communication with the target genes enriched by transcription factors through SCENIC (Methods; Figure 5C,E). Among them, the top three TFs were closely connected with multiple target genes in the regulons diagram: *SP6*, *JUND*, and *CEBPB*. These TFs were significantly activated in C0 and inhibited in other cell clusters; C3 was the most significantly inhibited (Figure 5C). Meanwhile, there were significantly up-regulated genes in C0 (Figure 5D).

## 3. Discussion

In recent years, intratumor genomic heterogeneity has been observed in various solid tumors. However, it is not clear how this heterogeneity is related to cancer progression. Our results showed that the PC-9s cells had population adaptation changes and could survive in the environment of hypoxia, low adsorption, and serum-free (SF) starvation. We also found evidence of LCSC heterogeneity in each different cell community, with each having different markers. This may reflect the true biological nature of the tumor, which should be analyzed at the single-cell level rather than the tumor bulk.

In this case, we are more concerned about how to block the adaptive viability of cancer cells. Through analyses, we identified the key factors and pathways associated with the anti-apoptotic and dedifferentiation ability of LCSCs. The results showed that oncoprotein *FOXM1* was the key switch in LCSC dedifferentiation (Figure 4D). Earlier studies have shown that *FOXM1* is involved in an early oncogenic pathway that predisposes cells to tumorigenesis by expanding the stem/progenitor cell compartment and deregulating subsequent keratinocyte terminal differentiation [25]. Furthermore, the overexpression of *FOXM1* leads to the acquisition of an EMT phenotype by activating the mesenchymal cell markers *ZEB1*, *ZEB2*, *Snail2*, E-cadherin, and vimentin, which are associated with increased spheroid-forming capacity and cancer stem cell surface markers (*CD44* and *EpCAM*) [26,27]. Therefore, the expression of *FOXM1* has become the focus of cancer testing and preventive drug development.

The Wnt/β-Catenin and NOTCH signaling pathways have been confirmed by many studies to be involved in the regulation of cancer stem cells, especially the mutual transformation with differentiated cancer cells [17,18]. The Wnt/β-catenin signaling pathway is an evolutionarily conserved signal transduction cascade and is mainly involved in cell proliferation, survival, movement, differentiation, and apoptosis [28]. However, the mechanism of transformation among cancer cells of different differentiation states leading to abnormal signal pathways is still unclear. By analyzing the cell–cell communication, we identified three TFs that are highly related to LCSC pathway genes (Figure 5D,E). It has been found that in the absence of SP6, the inner dental epithelium loses its characteristic ability to proliferate rapidly, resulting in severe enamel hypoplasia [29]. Therefore, SP6 is considered to be a multifunctional regulator of proliferation and differentiation, depending on the developmental stage. In addition, proto-oncogene *JUND* has been proposed to protect cells from p53-dependent aging and apoptosis [30]. Additionally, studies have confirmed that CEBPB plays an important role in regulating immune and inflammatory responses, among other processes [31,32]. Some studies have found that drug resistance to lung cancer is achieved through the synergistic effect of *NRF2* and *CEBPB* [33]. This evidence suggests that they may be key factors in the transformation among LCSCs of different differentiation states.

Intratumor heterogeneity means that different tumor cells within the same tumor may exhibit different genetic or phenotypic characteristics, which can be observed in patients with similar types of cancer [34]. Through flow cytometry and single-cell RNA-seq analysis, we found that compared with PC-9 cells, the expression of *CD44* increased in PC-9s cells, and the expression of *ALCAM* decreased, but the expression level of *ABCG2* was much lower (Figure 1B and S1D). To explore the factors underlying such transcriptomic characteristics, we grouped the LCSCs into four different clusters with different LCSC markers (*CD44*, *ABCG2*, and *ALCAM*) at the single-cell level. Many studies have found that lung cancer cells with a high expression of the *CD44* subset have higher tumorigenic ability, tumor sphere formation ability, and migration characteristics than those with low expressions of *CD44* [35,36,37,38]. Normal stem cells and LCSCs express high levels of ABC transporters, resulting in low intracellular drug concentrations [39]. Studies have shown that the *ABCG2* is highly expressed in LCSCs but is turned off in most terminally differentiated progeny [40]. The activated leukocyte cell adhesion molecule *ALCAM* was identified as an “inert” LCSC marker [41]. However, whether *ALCAM* can act as an LCSC marker has been controversial [42,43]. In earlier studies, the prognostic results based on the available LCSC markers were often inconsistent [22]; we found that the combined mode of three LCSC markers, i.e., *CD44*+*ABCG2*−*ALCAM*− or *CD44*−*ABCG2*+*ALCAM*−, especially the former, showed significant survival effects (Figure 3A,B). Compared with the *ALCAM*+ cell population, the *CD44*+ cell population was experimentally demonstrated to show a stronger association with gefitinib resistance.

It is undeniable there were some limitations to this study. First, we explored the features of LCSCs and their potential roles in LUAD heterogeneity based on PC-9 cells. Although PC-9 is a LUAD cell line and a small group of cells with stem cell characteristics has been detected, whether the results from this study can be observed in other cell lines should be investigated. Second, our analysis was mainly based on an in vitro cell line model of LUAD, which is unable to accurately model how LCSCs interact with all the molecules and cell types present in real tumors or organs. With the update in technology, understanding the dynamic mechanism between the LCSCs and tumor microenvironment will help to better understand the role of LCSCs in tumor genesis, growth, invasion, and therapeutic resistance.

## 4. Materials and Methods

The PC-9 cell line used in this study was provided by the Cell Bank of the Chinese Academy of Science (Shanghai, China). SPF female BALB/c-Nude mice (4–6 week old, 18–20 g) were purchased from Vital River Laboratory Animal Technology (Beijing, China). Epidermal growth factor (EGF) and basic fibroblast growth factor (bFGF) were purchased from PeproTech China (Suzhou, China). The human antibodies, *CD44*, *ABCG2*, *CD133*, *ALCAM*, and *EpCAM*, as well as Annexin-V apoptosis kits, were purchased from American BD (Pasadena, CA, USA). The DMEM/F12 medium, RPMI-1640 medium, TRIzol reagent, GIBCO^TM^ B27, and fetal bovine serum were purchased from Thermo Fisher Scientific China (Shanghai, China). Gefitinib was purchased from Sigma-Aldrich China (Shanghai, China). The CCK8 kits were purchased from Dojindo Laboratories China (Shanghai, China). Chromium Single Cell 3′ v3 Reagent Kit was purchased from 10× Genomics China (Shanghai, China). This study was approved by the Ethical Committee of Tianjin University General Hospital.

### 4.1. Serum-Free Cell Culture and Induced Differentiation of LUAD PC-9 Cells

A serum-free medium was used for cell culturing in this study, in which the stem cells could form cell spheres and maintain proliferation and differentiation potential [44]. Briefly, PC-9 cells in the logarithmic growth stage were placed in ultra-low absorption Petri dishes (10 ng/mL bFGF, 20 ng/mL EGF, 50 × B27) in a serum-free DMEM/F12 medium at the density of 5000 cells/mL, centrifuged, and the medium was changed every 3 days. Tumor cell spheres could be seen at 3–7 days. When reaching 20 cells/sphere, the cells were collected by centrifugation and washed with PBS once; then, the cells were continuously cultured in a serum-free medium. After the cell spheres were cultured for four generations for 7–10 days, the clones were inoculated into ultra-low adsorption Petri dishes at a cloning density of 1000 clones/mL. When the cells that formed cell spheres (referred to as PC-9s cells) were further cultured for 4 weeks, the cultures were centrifuged at 800 rpm for 5 min. Then, the supernatant was removed, and an RPMI-1640 medium containing 10% fetal bovine serum (without bFGF, EGF) was added, pipetted, and cultured in a 5% CO_2_ incubator at 37 °C and was observed under a microscope every 24 h. Cells were collected after one week of adherent culture.

### 4.2. Flow Cytometry and CCK8 Cytotoxicity Experiments

The spheres formed by normal and induced differentiated cells were transferred to and resuspended in a buffer (100 μL buffer per 10^7^ cells). To identify LCSCs, samples were analyzed by FACSAriaTM (BD) flow cytometry using *CD44*, *ABCG2*, *CD133*, *ALCAM*, and *EpCAM* human antibodies. Gefitinib was added to the logarithmic growth phase of PC-9 and PC-9s cells in a dose-dependent manner. After 48 h, 10 μL of the CCK8 reagent was added to each well, and the test was carried out 1 h later. Apoptosis analyses were performed with Annexin-V kits and operated according to the protocols. For each experiment, 3 biological replicates were included.

### 4.3. Animal Experiments and Tumor Specimens Collection

Animals were maintained in a temperature (22 ± 2 °C)- and humidity (50%)-controlled room on a 12 h light–dark cycle (lights on 7:00–19:00) with unlimited access to food and water. Twelve animals were randomly divided into four groups, with three animals in each group. For the first group, each animal was subcutaneously injected with 100 μL PC-9 cells at the right groin, with a concentration of 2 × 104/mL; for the second group, each animal was injected with 100μL PC-9 cells, with a concentration of 5 × 104/mL; for the third group, each animal was injected with 100μL PC-9s cells, with a concentration of 2 × 104/mL; for the fourth group, each animal was injected with 100μL PC-9s cells, with a concentration of 5 × 104/mL. After 1.5 months of inoculation, the nude mice were killed by the decapitation method, and the implanted tumors were carefully stripped before the volumes of the tumors were measured.

### 4.4. Bulk RNA Sequencing of PC-9 Cells and PC-9s Cells

The transcriptomics of PC-9 cells and PC-9s cells were measured by high-throughput bulk RNA sequencing. Briefly, the total RNA was extracted from PC-9 cells and PC-9s cells using a Trizol reagent according to the manufacturer’s instructions. Then, the prepared samples were sequenced by the BGI-Shenzhen (Shenzhen, China). The obtained paired-end reads of 150 bp were checked for quality via FastQC (v0.11.8). Salmon (0.8.0) was then adopted for quantification estimation based on the gene annotation for human genome build hg38 downloaded from GENCODE (release 28). 

### 4.5. Single-Cell RNA Sequencing of PC-9s Cells and Data Processing

10× Genomics single-cell RNA sequencing (scRNA-seq) was performed for PC-9s cells. Briefly, single-cell suspensions of PC-9s cells were prepared and processed as outlined by the 10× Genomics Single Cell 3′ v3 Reagent Kit user guide. cDNA libraries were prepared as recommended by the 10× Genomics v3 user guide with appropriate modifications to the PCR cycles based on the calculated cDNA concentration. The libraries were sequenced by BGI-Shenzhen.

Raw sequencing reads were aligned to reference the genome hg38 using CellRanger (version 3.0) to generate the raw gene expression matrices per sample. 

The feature-barcode gene expression matrix of untreated PC-9 cells using 10× genomics was obtained from the GEO dataset (GSE149383). Seurat R package (version 4.0.6) was used for downstream analyses [45]. Cells with <200 genes, >90% of the maximum number of genes, or >15% mitochondrial genes were filtered out. A total of 10,716 filtered PC-9 cells and 7320 filtered PC-9s cells were selected for analysis, respectively. The gene expression datasets were normalized using the “LogNormalize” method, and gene effects were removed by the ScaleData function. Subsequently, principal component analysis (PCA) was applied to identify the significant principal components, and 16 components were selected for uniform manifold approximation and projection for dimension reduction (UMAP) analysis. For PC-9s, the FindClusters function was used to classify the cells into 4 different clusters (C0, C1, C2, and C3) based on the gene expression profiles with a resolution of 0.18. The FindAllMarkers function with thresh.use = 0.25 and min.pct = 0.25 was applied to identify the differentially expressed genes (DEGs) in each cluster. In addition, the FindMarkers function was used to obtain DEGs in the designated two groups (C0 and C2) with the threshold of |log2FC| > 1.0 and *p*-value < 0.05. The batch effect was removed through the Seurat MultiCCA method, which employs a canonical correlation analysis (CCA) [46].

### 4.6. Bulk RNA Sequencing Data of Lung Adenocarcinoma (LUAD) Patients

The gene expression and clinical data corresponding to a total of 585 TCGA LUAD patients were obtained from UCSC Xena (http://xena.ucsc.edu/; accessed on 22 June 2022) [47]. The gene expression profile was measured by the format of log2(FPKM+1) values. DEGs were obtained using the R package “limma”.

### 4.7. EGFR Mutant LUAD Cell Lines and Analyses of the Intersected DEGs of TCGA LUAD and PC-9s

The gene expression profile of EGFR mutant LUAD cell lines and their IC_50_ (half-maximal inhibitory concentrations) of EGFR-TKIs, including gefitinib, erlotinib, afatinib, and osimertinib were obtained from GDSC2 (https://www.cancerrxgene.org/; accessed on 24 June 2022) [24]. To explore the performance of the intersected DEGs of TCGA LUAD (CD44+ABCG2-ALCAM- vs. CD44-ABCG2-ALCAM+) and PC-9s (C0 vs. C2) in the sensitivity of EGFR-TKIs, we calculated the Spearman correlation coefficients of each gene expression and EGFR-TKIs’ IC_50_. The overall survival plots were obtained for these DEGs and were analyzed by GEPIA2 (http://gepia2.cancer-pku.cn/; accessed on 25 June 2022): a tool for cancer and normal gene expression profiling and interactive analyses [48].

### 4.8. Survival Analysis

To determine the prognostic value of different LCSC clusters, Kaplan–Meier plots of the stem marker genes were used to explore the difference in overall survival (OS) and disease-free survival (DSS) among patients with low and high gene expressions grouped by the median. The R packages “survival” and “survminer” were used for this section, and the relationship was tested by the log rank.

### 4.9. Measuring the Stemness of PC-9 and PC-9s Cells by mRNAsi Scores

The degree of stemness for PC-9 and PC-9s cells was measured by mRNAsi: a predictive model to quantify the stemness of cancer stem cells by one-class logistic regression on the transcriptomic data of pluripotent stem cell samples (ESC and iPSC) [49]. The mRNAsi score ranged from 0 to 1, with higher values associated with biological processes active in cancer stem cells and greater tumor dedifferentiation. At the single-cell level, we calculated the mRNAsi scores of each PC-9 cell (GSE149383) and PC-9s cells based on their gene expression data obtained from scRNA-seq. Additionally, at the bulk transcriptome level, the mRNAsi scores of PC-9 and PC-9s cell lines were calculated through their RNA expression data (FPKM).

### 4.10. RNA Velocity

The “velocyto” Python tool recalculates spliced reads and unspliced reads based on the previously aligned bam files of scRNA-seq data for RNA velocity analysis [50]. Utilizing the “velocyto.R” and “Seurat” R packages, we determined the RNA velocity value for each gene in each cell and embedded the RNA velocity vector into the low-dimensional space. Using the gene.relative.velocity.estimates() function with the fit.quantile = 0.02 and kCells = 10, show.velocity.on.embedding.cor() with arrow.scale = 15 and *n* = 200, we projected the findings onto Umap.

### 4.11. Transcription Factor Enrichment through iRegulon

For the C2 cell cluster of PC-9s, there were cells developing from C0 and cells developing towards C0, which were recorded as “C0toC2” and “C2toC0”, respectively. The FindMarkers function was used to obtain DEGs in the two groups.

The iRegulon plug-in in Cytoscape software (www.cytoscape.org/; accessed on 27 June 2022) was used to predict transcription factors. The plug-in uses a normalized enrichment score (NES) to evaluate the reliability of prediction results. The greater the NES value, the higher the reliability. We used the DEGs just mentioned for transcription factor (TF) enrichment to obtain two groups of corresponding and predicted TFs. 

### 4.12. Cell–Cell Communication

Cell–cell interactions based on the expression of known ligand-receptor pairs in 4 different cell clusters were inferred using CellChatDB [51]. Simply said, we adhered to the official workflow, loaded the normalized counts into CellChat, and applied identifyOverExpressedInteractions and projectData with standard parameters set. Finally, netAnalysis_contribution, netAnalysis_signalingRole_network, and plotGeneExpression were used to calculate the contribution of each ligand-receptor to the overall signal pathway, determine the senders and receivers in the network, and display the expression of signal genes related to inferred import communications, respectively.

### 4.13. Identification of Key Transcription Factors in LCSCs

The cellular regulatory network was described by transcription factor (TF) analysis using SCENIC [52,53]. In short, scRNA-seq of PC-9s obtained from the previous pretreatment was used as the input. The matrix was then filtered with default parameters. The established gene regulatory network is shown in the heat map.

### 4.14. Gene Set Enrichment Analysis (GSEA)

Gene set enrichment analysis (GSEA; https://www.gsea-msigdb.org/gsea; accessed on 30 June 2022) is a computational evaluation used to evaluate whether the gene expression differences of biological samples between specific genomes are statistically significant [54]. GSEA GO BP was performed to analyze the DEGs in C2.

### 4.15. Gene Set Variation Analysis (GSVA)

GSVA is a GSE method that estimates the variation in pathway activity over a sample population in an unsupervised manner [55]. The activities in the Kyoto encyclopedia of genes and genomes (KEGG) and HALLMARK pathways were evaluated using the GSVA and GSVA package in R [55]. For the average expression of each PC-9s cell cluster, we summarized the expression-level rank statistics of a given pathway gene set into a final enrichment score (i.e., GSVA score), which was used to characterize the signature activity. Scores greater than 0 indicated an upregulation of pathway activity and vice versa.

### 4.16. Statistical Analyses

Statistical analyses in this study were performed via R version 4.0.2 (R Foundation for Statistical Computing; https://www.r-project.org/; accessed on 3 March 2022) and Python version 3.8 (https://www.python.org/; accessed on 16 March 2022). Package ‘Seurat’, ‘SCENIC’, ‘velocyto.R’, ‘CellChat’, ‘GSVA’, ‘tidyverse’, ‘ggplot2’, ‘GSEABase’ and ‘clusterProfiler’ of R, package ‘velocyto’, ‘pySCENIC’, ‘loompy’, ‘numpy’, and ‘scanpy’ of Python were mainly used for statistical and graphical analyses. *p*-value < 0.05 and |log2FC| > 1 was considered statically significant.

## 5. Conclusions

In conclusion, this study highlights the molecular composition and heterogeneity of PC-9 cancer stem cells, which can be used for LCSC targeting strategies. Based on single-cell transcriptome data, oncoprotein FOXM1 could be a key switch in LCSC dedifferentiation. In addition, the combined expression of CD44, ABCG2, and ALCAM was a specific marker for LCSCs. Patients with LUAD, especially CD44+ABCG2-ALCAM- have a worse prognosis. This provides new insights into intra-tumoral heterogeneity, tumor progression, and its impact on the clinical environment.

## Data Availability

The data are available upon reasonable request to the corresponding author.

## Supplementary Materials

The supporting information can be downloaded at: https://www.mdpi.com/article/10.3390/ijms24010482/s1.
