# Supplementary material for: Single-Cell Transcriptomics Unveils the Dedifferentiation Mechanism of Lung Adenocarcinoma Stem Cells
Source: Int J Mol Sci. 2022 Dec 28;24(1):482. doi: 10.3390/ijms24010482 (PMC9820263; doi:10.3390/ijms24010482)
Supplement: Supplementary file 1 [file ijms-24-00482-s001.zip › Supplementary Figures.pdf]

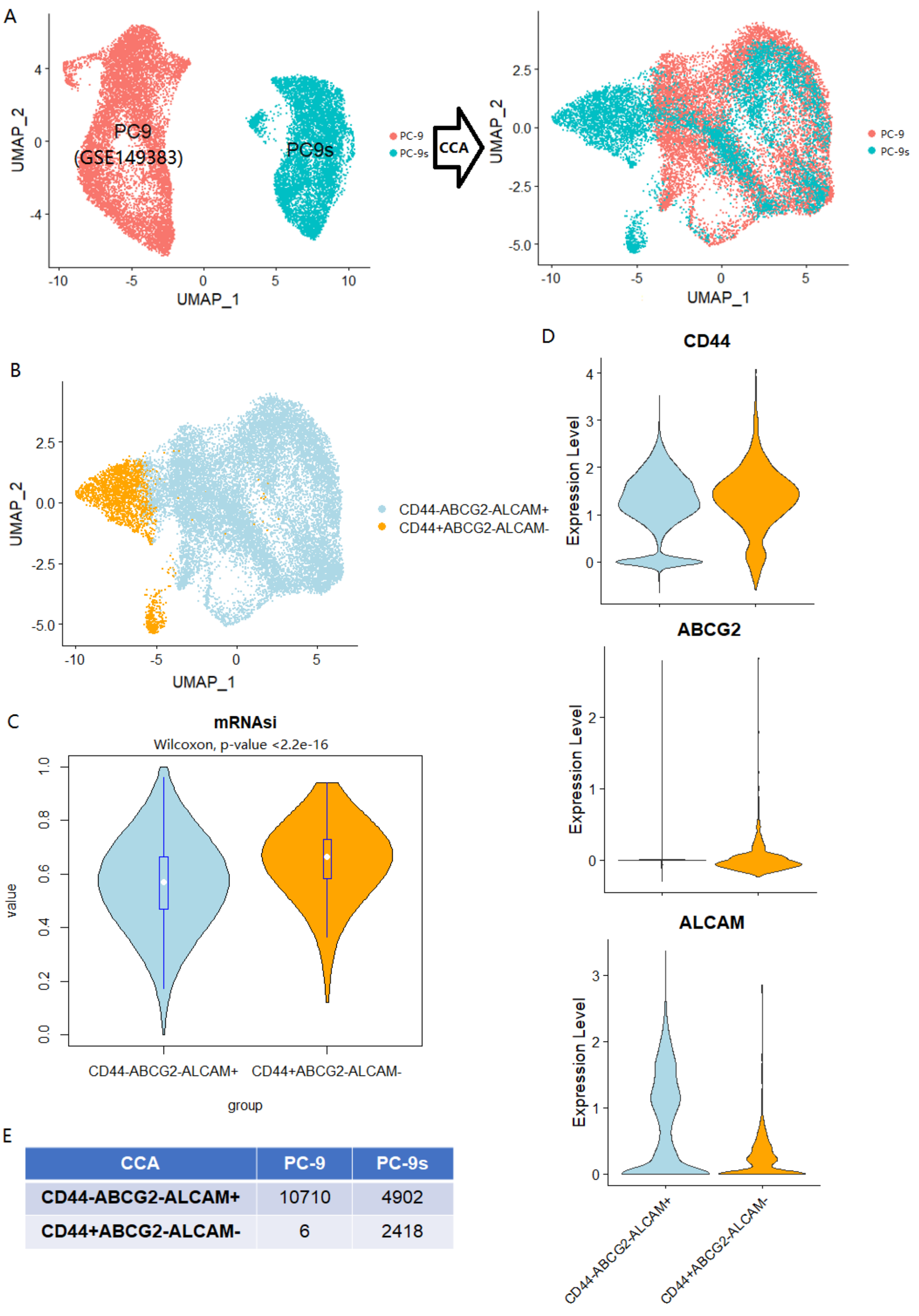

E

CCA	PC-9	PC-9s
**CD44-ABCG2-ALCAM+**	10710	4902
**CD44+ABCG2-ALCAM-**	6	2418

Figure S1. UMAP projection of PC-9s cells and PC-9 cells (GSE149383) before and after batch effect treatment (A). Based on expression of three cancer stem-like cell markers (CD44, ABCG2, ALCAM), the UMAP plot shows that the cell populations after batch effect treatment were divided into two groups: CD44+ABCG2-ALCAM-

and CD44-ABCG2-ALCAM+ (B). The mRNAsi scores of CD44+ABCG2-ALCAM- were significantly higher than CD44-ABCG2-ALCAM+ (Wilcox test, p-value <2.2e-16) (C). Violin plots display that expression of three cancer stem-like cell markers (CD44, ABCG2, ALCAM) between the two groups (D). The number of PC-9 cells and PC-9s cells in the two groups after batch effect treatment. There were 10,710 PC-9 cells and 4,902 PC-9s cells in CD44-ABCG2-ALCAM+, while CD44+ABCG2-ALCAM- only owned 6 PC-9 cells and 2,418 PC-9s cells (E).

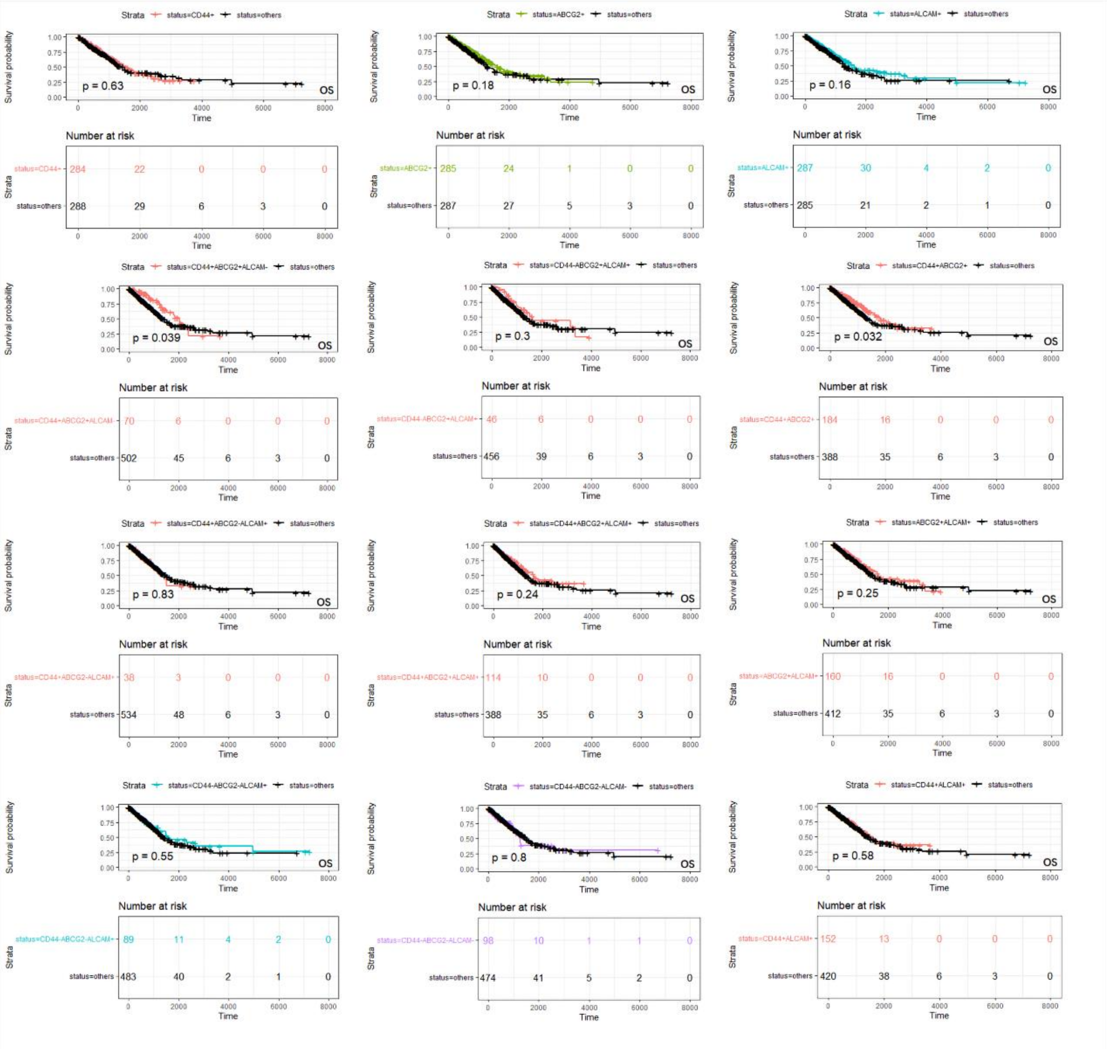

Figure S2. OS survival analyses of TCGA LUAD patients. High level (+) and low level (-) with the median of each marker gene expression across all patients as the threshold. Groups include: CD44+, ABCG2+, ALCAM+, CD44+ABCG2+ALCAM-, CD44-ABCG2+ALCAM+, CD44+ABCG2+, CD44+ABCG2-ALCAM+, CD44+ABCG2+ALCAM+, ABCG2+ALCAM+, CD44-ABCG2-ALCAM+, CD44-ABCG2-ALCAM- and CD44+ALCAM+.

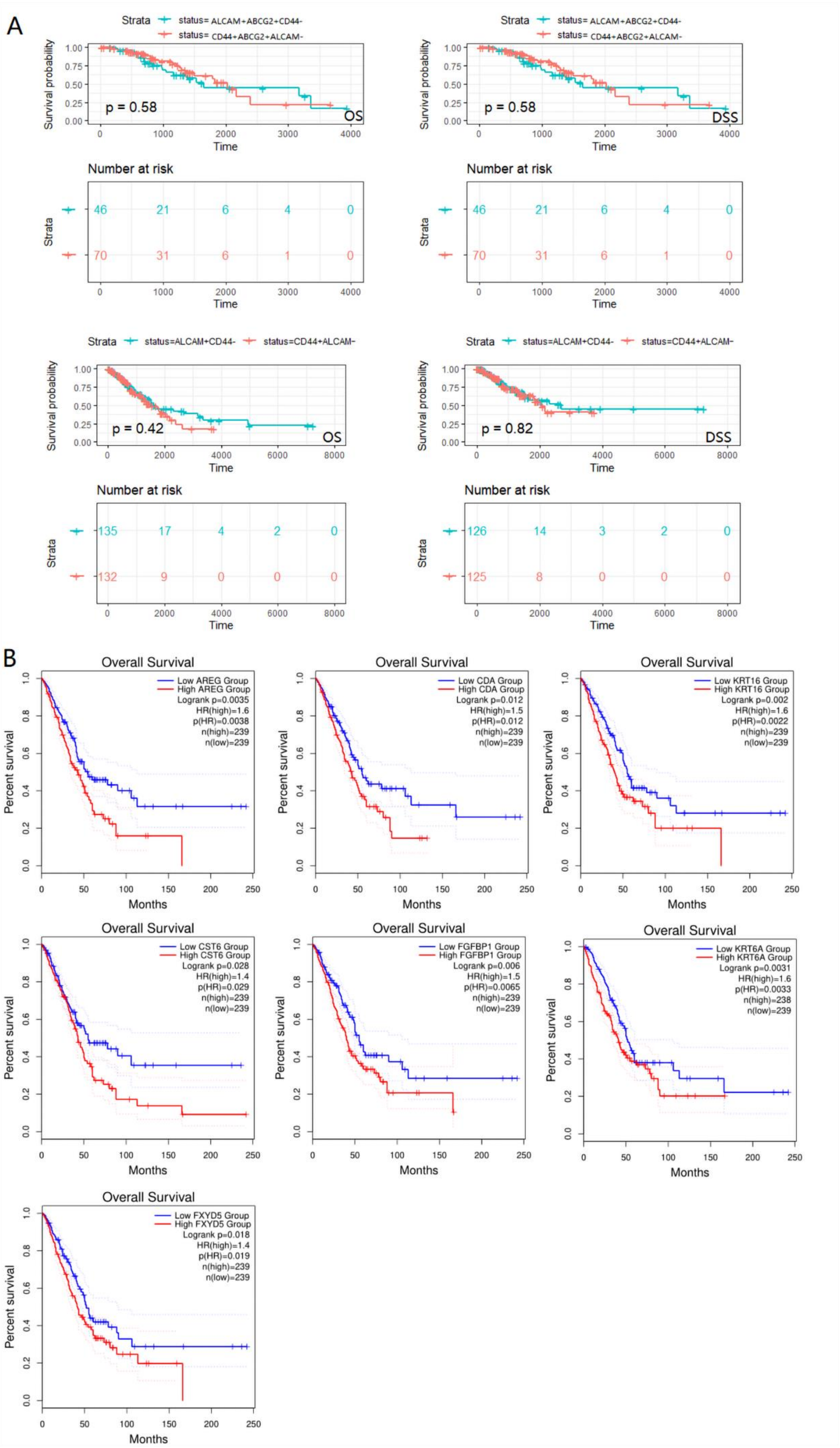
A
Strata
status= ALCAM+ABCG2+CD44-
status= CD44+ABCG2+ALCAM-
Survival probability
p = 0.58
OS
DSS
Time
Number at risk
46 21 6 4 0
70 31 6 1 0
status=ALCAM+CD44-
status=CD44+ALCAM-
p = 0.42
p = 0.82
135 17 4 2 0
132 9 0 0 0
126 14 3 2 0
125 8 0 0 0
B
Overall Survival
Percent survival
Months
Low AREG Group
High AREG Group
Logrank p=0.0035
HR(high)=1.6
p(HR)=0.0038
n(high)=239
n(low)=239
Low CDA Group
High CDA Group
Logrank p=0.012
HR(high)=1.5
p(HR)=0.012
n(high)=239
n(low)=239
Low KRT16 Group
High KRT16 Group
Logrank p=0.002
HR(high)=1.6
p(HR)=0.0022
n(high)=239
n(low)=239
Low CST6 Group
High CST6 Group
Logrank p=0.028
HR(high)=1.4
p(HR)=0.029
n(high)=239
n(low)=239
Low FGFBP1 Group
High FGFBP1 Group
Logrank p=0.006
HR(high)=1.5
p(HR)=0.0065
n(high)=239
n(low)=239
Low KRT6A Group
High KRT6A Group
Logrank p=0.0031
HR(high)=1.6
p(HR)=0.0033
n(high)=238
n(low)=239
Low FXYD5 Group
High FXYD5 Group
Logrank p=0.018
HR(high)=1.4
p(HR)=0.019
n(high)=239
n(low)=239

Figure S3. Survival curves (OS and DSS) of TCGA LUAD patients. High level (+) and low level (-) with the median of each marker gene expression across all patients as the threshold (A). The OS plots were obtained for intersected DEGs from table S3 were analyzed by GEPIA2 (http://gepia2.cancer-pku.cn/) (B).

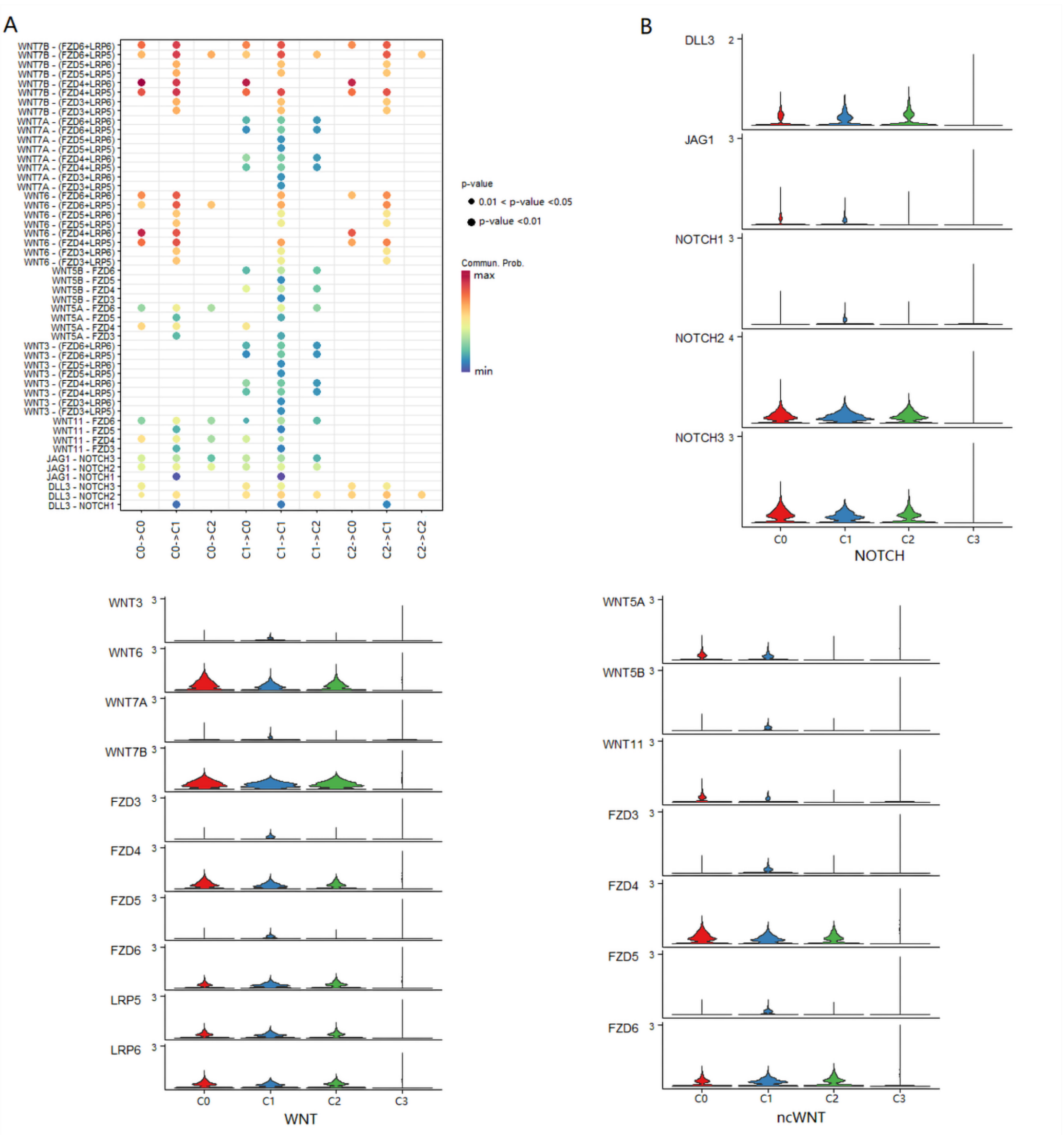

Figure S4. Bubble plot reveals contribution of each ligand receptor to the overall signaling pathway among three signaling pathways from figure 5A (p-value <0.05; A). Violin plots show the gene expression level related three signaling pathways in different cell clusters (B).
